# New Guinea bone daggers were engineered to preserve social prestige

**DOI:** 10.1098/rsos.172067

**Published:** 2018-04-25

**Authors:** Nathaniel J. Dominy, Samuel T. Mills, Christopher M. Yakacki, Paul B. Roscoe, R. Dana Carpenter

**Affiliations:** 1 Department of Anthropology, Dartmouth College, Hanover, NH 03755, USA; 2 Department of Biological Sciences, Dartmouth College, Hanover, NH 03755, USA; 3 Department of Mechanical Engineering, University of Colorado, Denver, CO 80217, USA; 4 Department of Anthropology, University of Maine, Orono, ME 04469, USA

**Keywords:** signalling theory, symbolic capital, bone mechanics, *Casuarius unappendiculatus*

## Abstract

Bone daggers were once widespread in New Guinea. Their purpose was both symbolic and utilitarian; they functioned as objects of artistic expression with the primary function of stabbing and killing people at close quarters. Most daggers were shaped from the tibiotarsus of cassowaries, but daggers shaped from the femora of respected men carried greater social prestige. The greater cross-sectional curvature of human bone daggers indicates superior strength, but the material properties of cassowary bone are unknown. It is, therefore, uncertain whether the macrostructure of human bone daggers exists to compensate for inferior material properties of human femora or to preserve the symbolic value of a prestigious object. To explore this question, we used computed tomography to examine the structural mechanics of 11 bone daggers, 10 of which are museum-accessioned objects of art. We found that human and cassowary bones have similar material properties and that the geometry of human bone daggers results in higher moments of inertia and a greater resistance to bending. Data from finite-element models corroborated the superior mechanical performance of human bone daggers, revealing greater resistance to larger loads with fewer failed elements. Taken together, our findings suggest that human bone daggers were engineered to preserve symbolic capital, an outcome that agrees well with the predictions of signalling theory.

## 1. Introduction

Signalling theory is a unifying concept in the social and biological sciences [1]. It proposes that social prestige, or symbolic capital [2], is a mechanism for communicating underlying traits with adaptive value. A central tenet of signalling theory is that status-accruing signals are honest (indexical) and, therefore, reliable indicators of the intrinsic qualities of the signaller. An intriguing application of signalling theory involves the decorative arts, a topic that is usually viewed as purely symbolic. Yet virtually every culture devotes effort to the elaboration of utilitarian objects (clothing, pots, tools, dwellings, etc.), as well as their own bodies. Universal behaviours invite an adaptive explanation [3], and signalling theory argues that symbolic expression can serve a functional purpose if it communicates, by proxy, attributes of the signaller, such as fine cognitive and motor skills or time available for non-subsistence behaviours. These qualities, in turn, are expected to attract higher quality allies and reproductive partners, thus enhancing the reproductive success of both the signaller and receiver.

**Figure 1.**
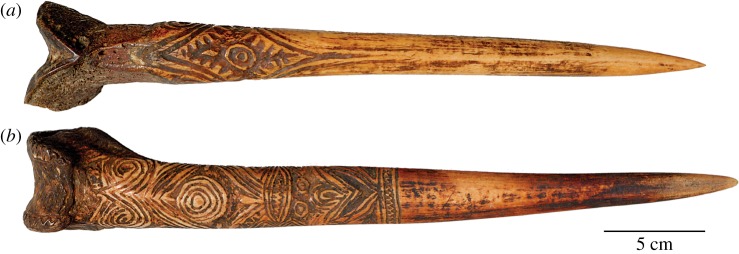
Bone daggers of the Sepik watershed, New Guinea. (*a*) Human bone dagger attributed to the Upper Sepik River (accession no. 990.54.28190.21, © Hood Museum of Art, Dartmouth College). (*b*) Cassowary bone dagger attributed to the Abelam people (accession no. 990.54.28190.12, © Hood Museum of Art, Dartmouth College).

**Figure 2.**
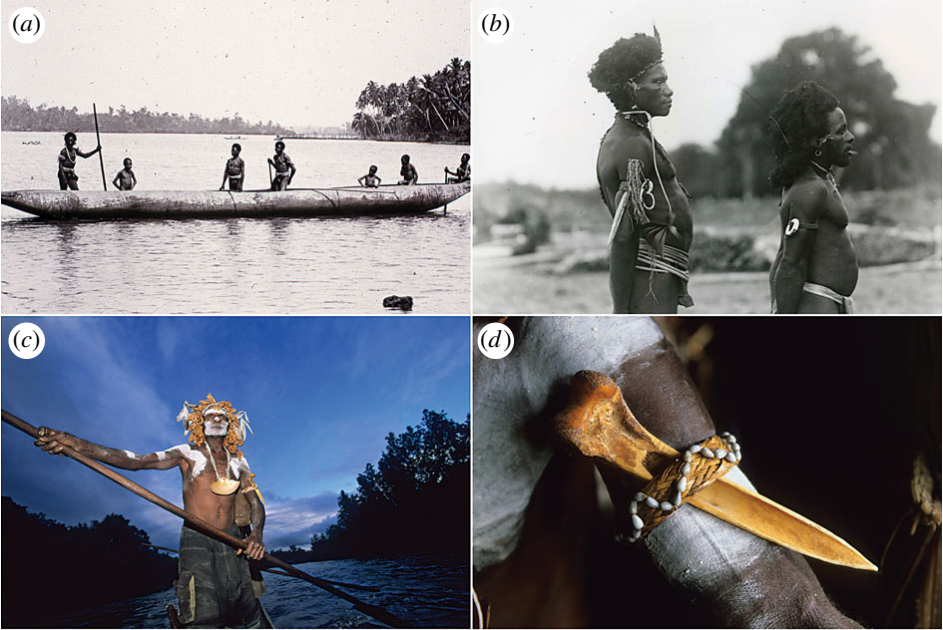
Anthropologist Albert Buell Lewis visited the Malol lagoon, Aitape, northern New Guinea in September 1909 [5]. (*a*) A bone dagger is attached to the left arm of the man in the centre of the canoe. (*b*) Armbands of woven rattan were used to secure daggers and other overt adornments, such as these shell rings. (*c*) The Asmat also produce bone daggers [6]; a cassowary bone dagger is attached to the left arm of an Asmat man punting a pirogue on the Seper River. (*d*) Posterior view reveals the removal of mediolateral cortical bone to create a flatter blade. Asmat photographs by Bruno Zanzottera, reproduced with permission.

The bone daggers of New Guinea were potent objects of artistic expression [4]. They were incised with elaborate designs, both abstract and representational (figure 1), and worn as conspicuous personal adornments (figure 2). It is a signalling tradition that invites study because, as close-combat weapons [4,7], bone daggers were exemplars *par excellence* of male fighting abilities, and a highly desirable status symbol among men [8,9]. In addition, bone itself was the embodiment of strength, both mechanically and symbolically with powers enmeshed in the supernatural world [4]. This dual concept of strength, together with the dual function of bone daggers as weapons and symbols, is intriguing when one considers the different macrostructures of bone daggers. Those made from a human femur were shaped differently and appear to be better engineered for mechanical performance, which raises the possibility of biomechanical trade-offs in a social signal, a topic that intersects the arts with the physical, life and social sciences.

### 1.1. Bone daggers as weapons

In New Guinea, bone daggers were close-combat weapons used to kill outright or finish off victims wounded with arrows or spears, by stabbing them in the neck ([8–11]; Bragge LE. n.d. (1970–1974) *Interview notes* (unpublished). Koetong, Australia: Bragge Archives.) a process that Schultze Jena [12] described vividly in 1914:

The lethal point at which one aims [the bone dagger] is the neck just above the breastbone end of the collarbone, the area of the subclavia and carotid. The dagger serves not only to stab into the main arteries but at the same time as a lever with which one twists the punctured neck of the enemy in order to tear the throat and, with sufficient power, break the neck (p. 9; German–English translation by P. Roscoe).

Landtman published a similar account based on interactions with the Kiwai from 1910 to 1912 [13]. According to Kiwai respondents, the eastern Gulf tribes used bone daggers for ‘stabbing prisoners, taken in a fight, through their hip joints, knees or ankles’. Thus disabled, the prisoners ‘could be kept alive until needed for a later cannibal feast’ (p. 57).

**Figure 3.**
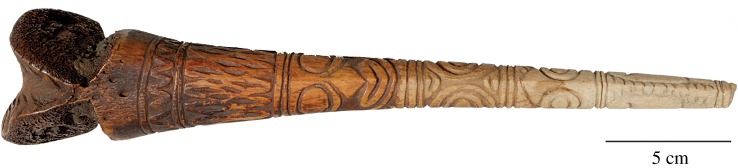
Mechanical failure near the tip is evident in some bone daggers. This human bone dagger is attributed to the Kwoma people, with affinities to the Iatmul area of the Middle Sepik River (early- or mid-twentieth century; accession no. 990.54.28190.11, © Hood Museum of Art, Dartmouth College).

The veracity of these accounts is difficult to assess; contact-era narratives were often based on the views of informants toward their own adversaries. However, the reports are consistent insofar as they describe stabbing actions in various joints (cervical, hip, knee, ankle). Another similarity is implicit, and it concerns compressive and torsional loads near the tip and the potential for mechanical failure, which is evident in some specimens (figure 3). Such failure would empty the dagger of all symbolic strength and potentially jeopardize the user during hand-to-hand fighting, suggesting that the strength of daggers can hold adaptive value. It is telling that peacemaking ceremonies in the Lower Arafundi require the mutual destruction of spears but the exchange of bone daggers [14], a distinction that highlights the practical value of the latter weapon.

### 1.2. Bone daggers as objects of social prestige

The biological source of a bone dagger (human or cassowary) is readily apparent [4]. Those shaped from human femora have distinct pommels (notched femoral condyles and a steep patellar groove; figure 1*a*) and greater curvature, both longitudinally and transversely. Human bone daggers were prestigious [4] in part because the most suitable femora were sourced from battle-proven men, usually those of a father, once the corpse was reduced to a skeleton [4], or those of a vanquished enemy [15]. They were weapons filled with substantial strength, i.e. they were the manifestation of spiritual power [16], allowing the owner to lay claim to the powers of the man who surrendered the bone.

**Figure 4.**
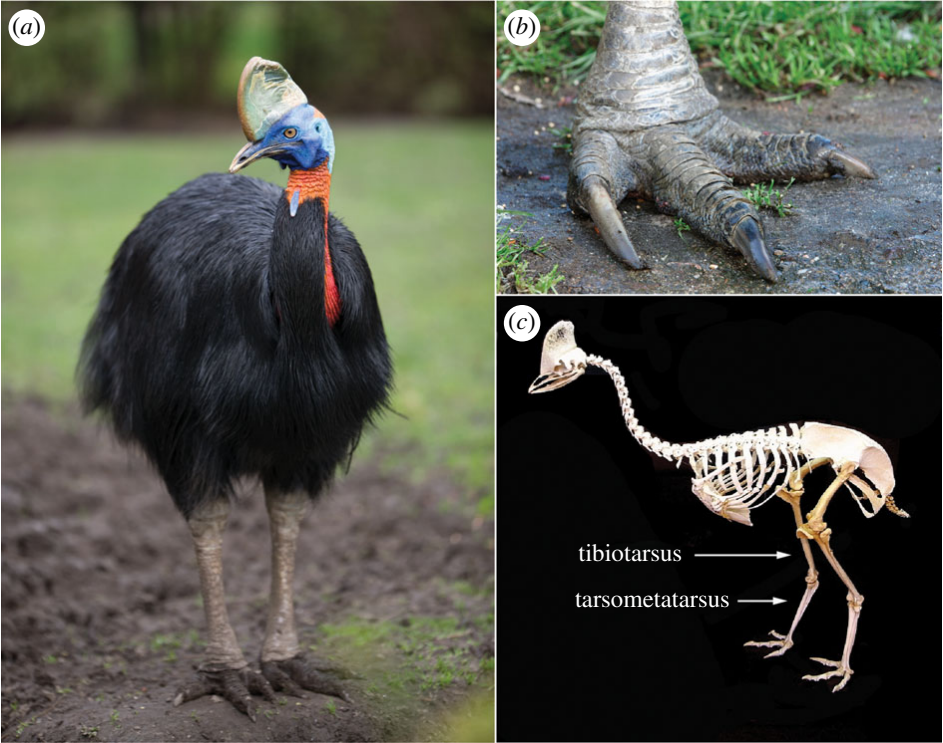
(*a*) Northern or single-wattled cassowary (*Casuarius unappendiculatus*); females can stand 2 m tall and weigh up to 58 kg (photograph by Holger Ehlers, reproduced with permission). (*b*) Cassowaries have stout legs with dense, apneumatic bones and large, three-toed (tridactylus) feet [17]. Massive leg muscles [18] enable running speeds up to 50 km h^−1^ and standing jumps as high as 1.5 m [19]. An outstanding peculiarity of cassowaries is their medial toe (digit II), which is equipped with a prodigious spike-like claw (photograph by Christian Hütter, reproduced with permission). The claw can be 12 cm long and 3 cm at the base [20] and used to telling effect by quickly extending (kicking) the leg forward or to the side. A review of 221 incidents between 1926 and 1999 found that southern cassowaries (*C. casuarius*) inflicted serious wounds on domestic animals, with kicks resulting in lacerations, punctures, and ruptures to internal organs [21]. At least one person, a boy aged 16 years, stumbled and fell while assailing a cassowary and later succumbed to an exsanguinating puncture wound to the neck [22]. (*c*) Articulation of the tibiotarsus and tarsometatarsus at the intratarsal joint. The area that appears to be the knee of a bird is homologous to the human ankle.

**Figure 5.**
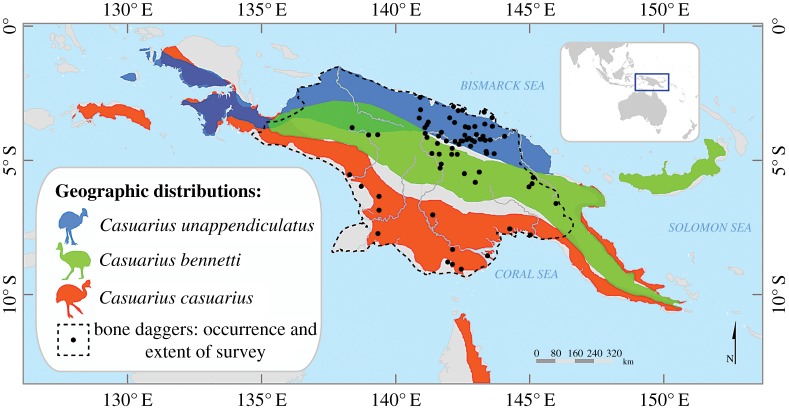
Geographical ranges of cassowary species: the northern or single-wattled cassowary (*C. unappendiculatus*); the Bennett's or dwarf cassowary (*C. bennetti*); the southern or double-wattled cassowary (*C. casuarius*) [23]. Points depict the recorded occurrence of cassowary bone daggers; the hashed line depicts the limits of our ethnographic survey (P. Roscoe 2017, unpublished dataset).

Daggers were also shaped from the tibiotarsus of cassowaries (figure 4), and these were widespread (figure 5), especially in the Sepik region [24]. There is reason to surmise that cassowary bone daggers were also symbols of male strength, although to lesser extent [4]. Cassowaries are described as ‘sullen, treacherous and extremely pugnacious' [25], words that speak to their size, agility and aggression when provoked (figure 4). Hunting such an animal was an additional source of male status in New Guinea [26,27]; indeed, some odes to deceased men recounted the number of his cassowary kills [28]. Cassowary bone daggers also featured prominently in local prestige economies [29,30], in part because cassowaries were imbued with deep cultural significance: commonly sexed as female, they were widely metaphorized and mythically and ritually cast as women, wives and sometimes enemies rather than as birds [31–34]. Possession of a cassowary bone dagger was thus a plausible signal of male hunting ability, physical and ritual strength and status.

Another explanation for the prevalence of cassowary bone daggers is more utilitarian: it might have outstanding mechanical properties. When compared to mammals, the mass of compact (cortical) bone in birds is distributed relatively far from the long axis, leading to higher second and polar moments of area and greater inferred resistance to bending and twisting [35], ideal properties for any tool. It is perhaps unsurprising that the tibiotarsius and tarsometatarsus of cassowaries (figure 4*c*) were often fashioned into practical tools, e.g. coconut splitters [36,37] and pandanus splitters [38–41]. Indeed, the mechanical strength and dagger-like appearance of these implements has led some authors to suggest that early accounts misidentified bone ‘daggers’ as weapons rather than tools. However, this is not the case: the tips of these tools are usually blunted (see electronic supplementary material, figure S2), whereas true bone daggers were sharpened to a fine point (cf. figures 1 and 2*d*).

In sum, cassowary bone appears superficially to have a similar mechanical utility for dagger manufacture to human bone, yet human bone daggers have greater prestige than those of cassowary bone. These differences focus the aims of the present study.

### 1.3. Study aims

Bone daggers were ornaments and armaments, and the retention of greater cross-sectional curvature appears to be a deliberate design feature of all human-derived daggers. It is a difference that, *a priori*, would suggest better mechanical performance. Yet the material properties of cassowary bone daggers are unknown, and the widespread use of ratite leg bones—e.g. moa bone daggers in prehistoric New Zealand [23] and emu bone daggers in Australia [42,43]—raises the possibility that cassowary bone has ideal material properties. It is, therefore, uncertain whether the superior macrostructure of human bone daggers exists to compensate for inferior material properties or to better preserve an object with greater symbolic capital. Here we test between these competing possibilities.

## 2. Material and methods

### 2.1. Specimens and imaging

We examined intact bone daggers accessioned in the Hood Museum of Art, Dartmouth College. This sample includes early- and mid-twentieth century specimens derived from human femora and cassowary tibiotarsi (*n* = 5 each; table 1). In addition, we purchased a modern (*ca.* 1970s) cassowary bone dagger from a private art dealer (see Ethics statement). The human-derived daggers in our sample are readily distinguished by their greater curvature and richer patina. They are also rather rare. An unpublished survey of museum collections (those of the American Museum of Natural History; the Field Museum of Natural History; the Peabody Museum of Archaeology and Ethnology; and others) found that 21 of 499 bone daggers (4.2%) were shaped from human femora (M. Golitko 2017, personal communication).

**Table 1.** Museum-accessioned bone daggers examined in the present study. The daggers were acquired by Harry A. Franklin (1904–1983), Los Angeles, California, in the 1950s; bequeathed to the Harry A. Franklin Family Collection, Los Angeles, California, in 1983; and gifted to the Hood Museum of Art, Dartmouth College, Hanover, New Hampshire, in 1990.

accession no.	bone source^a^	known provenance; cultural attribution^a^
990.54.27734	cassowary	Ndu language family
990.54.27742	cassowary	Sepik River, Abelam people
990.54.27743	human	Middle Sepik River, Kwoma people
990.54.27751	cassowary	Sepik River, Abelam people
990.54.28190.7	human	Kwoma people (likely)
990.54.28190.9	human	Upper Sepik River
990.54.28190.10	cassowary	Abelam people
990.54.28190.12	cassowary	Abelam people (likely)
990.54.28190.16	human	Kwoma people (likely)
990.54.28190.21	human	Upper Sepik River

^a^Affirmed in 1992 by Douglas Newton, Metropolitan Museum of Art, and Kathleen Barlow, Central Washington University.

**Figure 6.**
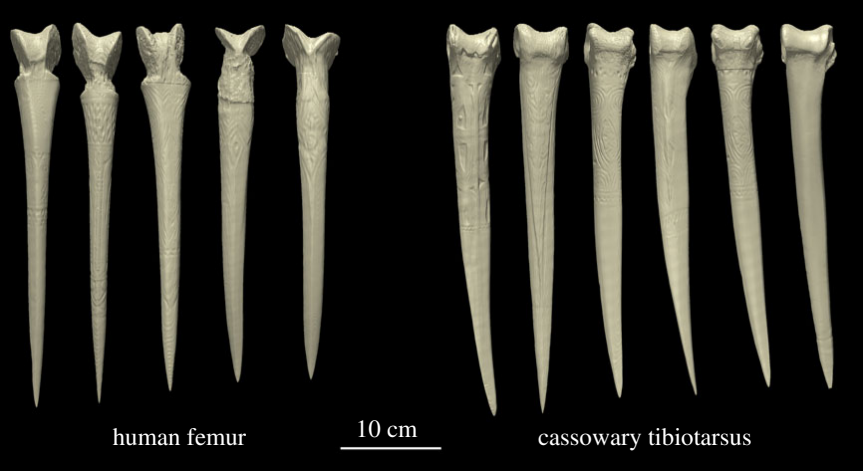
Three-dimensional reconstructions of each dagger in the present study. This grouping of (*a*) human-derived daggers and (*b*) cassowary-derived daggers is useful for highlighting differences in the pommels, which stem from anatomical differences in the human knee joint and cassowary intratarsal joint (figure 4*c*). The distal condyles of the human femur are asymmetrical, and the patellar groove is much steeper than the shallow trochlear surface of the distal tibiotarsus of cassowaries. In human bone daggers, much of the medial and lateral epicondyles of the femur were removed to create a steeply notched, V-shaped pommel.

**Figure 7.**
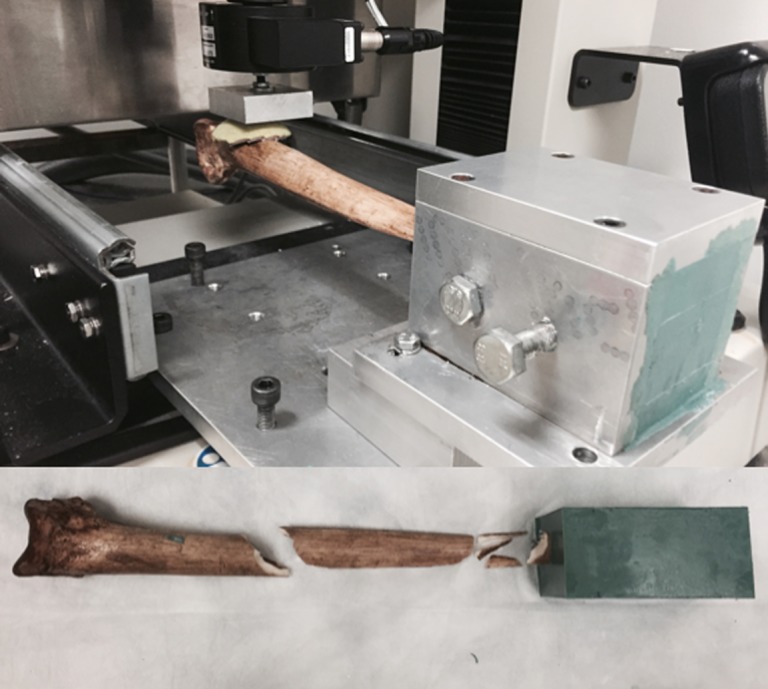
Cantilever bending test configuration; the cassowary bone dagger was fixed at 20% of the overall length from the tip.

We scanned each specimen in a 16-slice spiral computed tomography (CT) system (LightSpeed 16, General Electric Medical Systems, Milwaukee, WI, USA) located in the Department of Radiology, Dartmouth-Hitchcock Medical Center. We used a voxel size of 0.2 × 0.2 × 1.25 mm to create three-dimensional reconstructions of each dagger (figure 6) and we measured the length (*L*) of each dagger on the basis of these images. We estimated dagger penetration in human joints at 20% of the overall length (figure 7), as measured from the tip. We, therefore, used this distance to compare cross-sectional geometries. We used BoneJ [44] to measure geometrical properties, including cross-sectional area (CSA), minimum and maximum moments of inertia (*I*_min_, *I*_max_—measures of bending resistance; see electronic supplementary material, figure S2), minimum and maximum section moduli (*Z*_max_, *Z*_min_—proportional to bending strength) and polar section modulus (*Z*_p_—proportional to torsional strength).

### 2.2. Cassowary bone material properties

To measure bone properties and dagger strength, we used a modern cassowary bone dagger for CT imaging and destructive testing. It was first tested in cantilever bending using a uniaxial mechanical tester with a 500 N load cell (Insight 30, MTS, Eden Prairie, MN, USA; figure 7). To simulate insertion into a human joint, we embedded 20% of the dagger length into urethane casting material (DynaCast, Freeman Manufacturing and Supply, Avon, OH, USA). Then we inserted a small sheet of rubber between the compression platen and bone in order to apply load evenly to the dagger handle. We loaded the dagger in the anteroposterior direction to failure at a displacement rate of 1 mm s^−1^. We used the maximum force measured in this test (see electronic supplementary material, figure S3) as a benchmark to establish a failure criterion for the finite-element (FE) modelling of each dagger.

**Figure 8.**
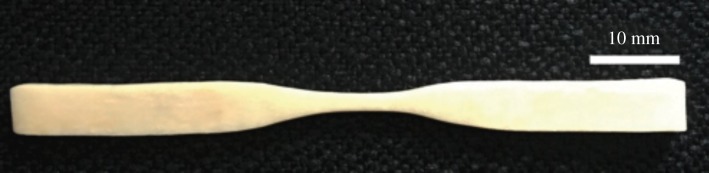
Example dog-bone preparation for mechanical testing. Scale bar, 10 mm.

We used excess pieces of the dagger to create three dog-bone samples for mechanical testing (figure 8). The samples were taken from the mid-diaphysis of the bone, and were cut and sanded to a uniform cross-section with no curvature. The samples were tested to failure in tension using a 30 kN load cell at a displacement rate of 0.01 mm s^−1^. We used uniaxial strain gages (L2A-06-062LW-120, Micro-Measurements, Vishay Measurements Group, Raleigh, NC, USA) to measure strain at the narrow section of each sample. After testing, we measured the CSA at the failure location using a flatbed scanner at a resolution of 0.01 mm pixel^−1^. We used the resulting area to convert force measurements to equivalent stress. We calculated the Young's modulus (*E*) of each sample using the slope of the linear portion of the stress–strain curve, and defined the ultimate stress (*σ*_ult_) as the highest stress achieved during a test.

### 2.3. Finite-element model and analysis

We converted CT scans into FE models using ScanIP + FE software (Simpleware, Synopsys, Mountain View, CA, USA). We used FE modelling because it converts complex structures into simpler, smaller sections for mechanical analysis and because it is non-destructive. FE models have become a powerful tool for evaluating the mechanical performance of osteological and fossil specimens in museum collections [45–50]; however, the focus of these ‘osteometric eyes’ [50] is seldom turned on objects of art or material culture in the ethnographic or archaeological records (but see Thomas *et al*. [51] and their FE analysis of fluting in North American Pleistocene weaponry).

We segmented images using a threshold-driven region-growing algorithm, and meshed each model with linear four-node tetrahedral elements (average number of elements = 88 110). The number of elements was determined to be sufficient after a convergence study found no significant increase in the accuracy of the models with more elements. We separated trabecular bone from cortical bone using the grey-scale value of the CT scans for each voxel, where the trabecular bone could then be assigned material properties. Trabecular bone was confined to the grip and pommel regions of each dagger; i.e. the distal condylar region of the human femur or cassowary tibiotarsus. Our mechanical tests suggest that the Young's modulus of cassowary and human cortical bone [52–57] are practically equivalent (see Results and discussion). Accordingly, we used identical material properties for all models, assigning a Young's modulus of 24.0 GPa to cortical bone (present results), a Young's modulus of 0.4 GPa to trabecular bone [58] and a Poisson's ratio of 0.3 to all bone tissue.

We imported the FE models into ABAQUS (Simulia, Dassault Systèmes, Waltham, MA, USA) for analysis. We fixed each model at 20% of the length measured from the tip, and applied a 225 N force to the handle end. The tested dagger failed at 200 N, but a slightly higher force of 225 N was applied to the FE models to ensure failure. Bending was performed in two perpendicular directions (corresponding to *I*_max_ and *I*_min_) in order to estimate the maximum and minimum bending strength values of each dagger. We imported the von Mises stress distribution of each dagger into Matlab (Mathworks, Natick, MA) to determine failure load, which we estimated based on a per cent volume failure criterion. In the model of the cassowary dagger used for testing, the von Mises stress in 3.3% of the overall volume exceeded the *σ*_ult_ of bone tissue at the measured failure load. Given these results, we used a 3.3% volume criterion for all models. We used the FE-based stress distribution for each dagger and scaled the applied load until 3.3% of the volume exceeded *σ*_ult_. This method to calculate the failure force allows for any force value to be applied to the models, and the correct failure force will still be calculated. The resulting force (*F*_max_) is assumed to be the force required to induce fracture. For additional comparison, we calculated the forces required to induce failure in 1%, 3% and 5% of the volume of each dagger.

### 2.4. Statistical analysis

We used Mann–Whitney U tests to compare the means of all geometric properties and FE-predicted failure loads in our sample of five human bone daggers and six cassowary bone daggers, where the sixth cassowary bone dagger was purchased and used for mechanical testing. Statistical differences were set at *α* < 0.05.

## 3. Results and discussion

Ethnographic accounts of bone daggers report that users targeted the cervical vertebrae or the hip, knee and ankle joints of their victims. We estimated that penetration of these joints would entail 20% of the length of dagger; therefore, this distance, as measured from the tip, was used to compare the cross-sectional geometries of human- and cassowary-derived daggers. CT images affirmed our subjective impressions of greater cross-sectional curvature among the human bone daggers, a shape that predicts superior mechanical performance (see electronic supplementary material, figure S2). Indeed, *I*_min_ differed between the two dagger types (table 2), with the mean value of human bone daggers being 290% greater than that of cassowary bone daggers despite similar cross-sectional areas (table 2). Thus, the retention of greater cross-sectional curvature during the manufacture of human bone daggers appears to be a design feature that results in higher moments of inertia and greater resistance to bending; i.e. a stronger dagger.

**Table 2.** Mean (±1 s.d.) geometric and mechanical parameters for the human and cassowary bone daggers in our sample. Moment of inertia: resistance to bending (dependent on bending direction). Section modulus: directly proportional to bending strength (dependent on bending direction). Polar section modulus: Directly proportional to torsional strength.

measured parameters	human bone daggers (*n* = 5)	cassowary bone daggers (*n* = 6)
length (mm)	358.2 ± 19.6	374.9 ± 16.7
cross-sectional area (mm^2^)	87.0 ± 25.7	78.4 ± 9.3
min. moment of inertia, *I*_min_ (mm^4^)	607.1 ± 393.0*	206.7 ± 73.2*
max. moment of inertia, *I*_max_ (mm^4^)	1762.4 ± 945.0	1639.1 ± 328.1
min. section modulus, *Z*_min_ (mm^3^)	98.3 ± 47.6	59.5 ± 15.2
max. section modulus, *Z*_max_ (mm^3^)	225.7 ± 99.7	182.4 ± 23.6
polar section modulus, *Z*_p_ (mm^3^)	253.7 ± 118.9	193.7 ± 26.5
finite-element breaking force, *I*_min_ (N)	462.6 ± 131.6**	159.5 ± 29.3**
finite-element breaking force, *I*_max_ (N)	455.2 ± 104.4*	327.8 ± 54.2*

*p < 0.05.

**p < 0.01.

To explore whether the superior macrostructure of human bone daggers exists to compensate for the inferior material properties of human bone, we tested the mechanical properties of a cassowary bone dagger to establish baseline properties for FE models. Flexural strength in cantilever bending was 200 N, and corresponded to testing in the *I*_min_ direction. Tensile tests revealed a mean (±1 s.d.) elastic modulus (*E*) of 24.01 ± 1.57 GPa and an ultimate stress (*σ*_ult_) of 153.9 ± 42.3 MPa. This finding compares well with measures from an ostrich (*Struthio camelus*; *E*: 13.90 GPa [55]) and emu (*Dromaius novaehollandiae*; *E*: 13.05 ± 3.94 GPa, range: 5.62 to 19.83 GPa; *σ*_ult_: 146 MPa [59]), with the caveat that these authors examined the compact bone of fresh (undried) femora. In our tests, the stress–strain behaviour of dry, tibiotarsal compact bone was relatively linear-elastic, showing a brittle response and failure before 1% strain (see electronic supplementary material, figure S4). Crucially, our measures of material properties fall squarely between (*E* = 18.0 and 27.4 GPa [53,58]) and overlap (*σ*_ult_ = 103–133 MPa [52–57]) published values for dry compact bone from human femora, which simplified the construction of our FE models.

**Figure 9.**
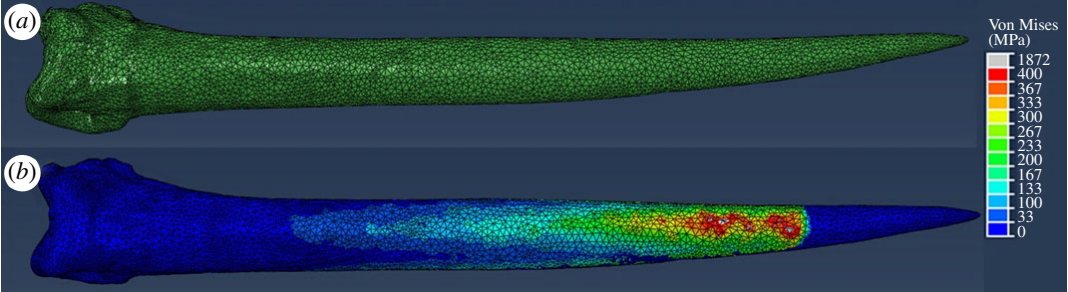
(*a*) Example of mesh density in the FE model. (*b*) Example stress distribution during a simulated cantilever bending test.

**Figure 10.**
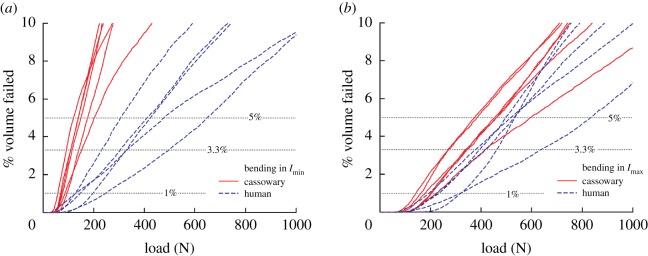
Per cent volume of failed elements as a function of simulated bending models in the (*a*) *I*_min_ and (*b*) *I*_max_ directions.

FE models of the daggers underwent simulated cantilever-bending experiments to analyse the failure loads of each dagger (figure 9). First, an FE model of the experimental bending test was subjected to 200 N (i.e. the flexural strength of the dagger). The simulation showed that 3.3% of the volume of the dagger had a stress greater than the *σ*_ult_. Next, FE models of all daggers were subjected to simulated bending tests. The influence of force on the percentage of volume failed in the daggers was compared in bending with respect to *I*_min_ and *I*_max_ (figure 10). All daggers followed the same general trends. At low loads (less than 50 N) the stresses in the models remained below the *σ*_ult_ and thus no volume of the model was considered ‘failed’.

**Figure 11.**
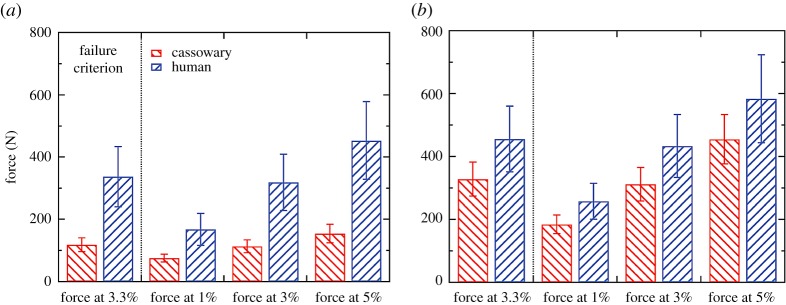
Mean forces (±1 s.d.) required to reach a critical volume of failed elements in the (*a*) *I*_min_ and (*b*) *I*_max_ directions.

With increasing load, stresses in the model increased and the volume of failed elements increased in a linear fashion. When testing in the *I*_min_ direction, each cassowary model demonstrated less force was required to induce failure for a given percentage of volume; however, the cassowary daggers showed more similar behaviour to the human daggers when tested in the *I*_max_ direction. We compared the failure load of the daggers at 1.0, 3.0, 3.3 and 5.0% of the failed volume of elements, where 3.3% corresponded to the volume of failed elements for the dagger tested experimentally (figure 11). When tested in the *I*_min_ direction, the human bone daggers were significantly stronger than the cassowary bone daggers at all levels of failed volume (table 2). For example, the human bone daggers required 254 N to fail at 3.3% of the total volume of the dagger, which corresponds to 31% more force, on average, compared to the cassowary bone daggers. However, when tested in the *I*_max_ direction, the human bone daggers required only 27% more force on average.

## 4. Conclusion

Our results suggest that the mechanical properties of compact bone are similar in the human femur and cassowary tibiotarsus, although our analysis is limited by necessity to a single sample of cassowary bone. Still, this finding suggests that systematic differences in the macrostructure (cross-sectional curvature) of bone daggers will determine differences in mechanical strength. We affirmed this prediction with FE models, finding that human bone daggers can support larger loads with a smaller volume of elements failing. We conclude by suggesting that the retention of greater diaphyseal curvature is a deliberate design feature intended to produce a stronger bone dagger.

It is, therefore, difficult to explain why dagger-makers working with a tibiotarsus would choose to remove so much of the mediolateral wall (figure 2*d*). A flatter cross-sectional shape is a weaker macrostructure and it is tempting to speculate that the disadvantages of this design are balanced by greater comfort for the owner (when fixed to the upper arm; cf. figure 2) or perhaps reduced weight during fighting or friction during insertion. In the event of breakage, a cassowary bone dagger is easily replaced, whereas a human bone dagger is not. We conclude by suggesting that people in the Sepik region of New Guinea engineered human bone daggers to withstand breakage, and that their prevailing motivation was to preserve intact the embodiment of symbolic strength and social prestige, an outcome that agrees well with the predictions of signalling theory.

## Supplementary Material

Figure S1

## Supplementary Material

Figure S2

## Supplementary Material

Figure S3

## Supplementary Material

Figure S4

## Data accessibility

Three-dimensional meshes were deposited in the MorphoSource data archive (http://morphosource.org; media nos. 16844–16859); corresponding data on the geometry and mechanical parameters of each specimen were deposited in the Dryad Digital Repository (http://dx.doi.org/10.5061/dryad.f7p1n17) [60].
