# Supplementary figures and images for: New Guinea bone daggers were engineered to preserve social prestige
Source: R Soc Open Sci. 2018 Apr 25;5(4):172067. doi: 10.1098/rsos.172067 (PMC5936927; doi:10.1098/rsos.172067)

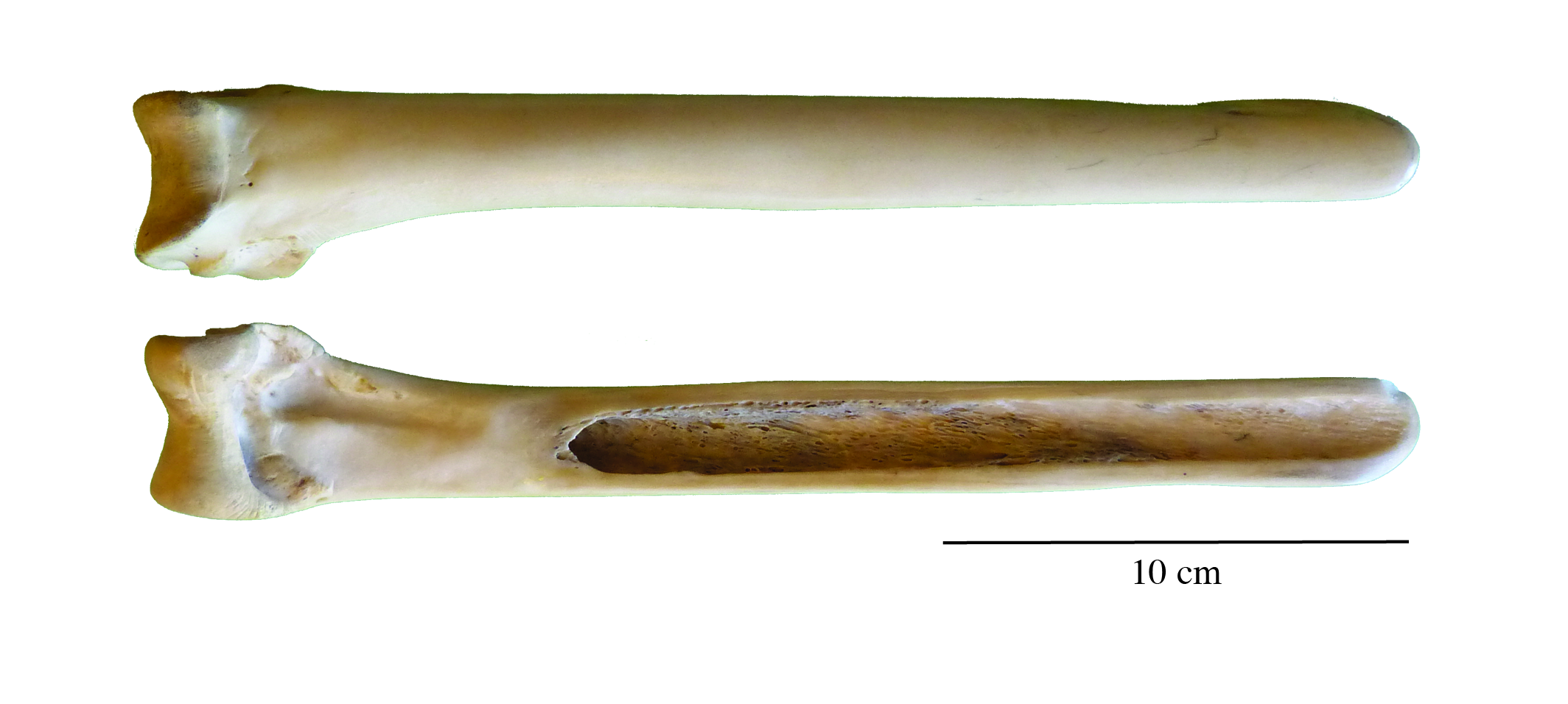

Supplement: Figure S1 [file rsos172067supp1.tif]

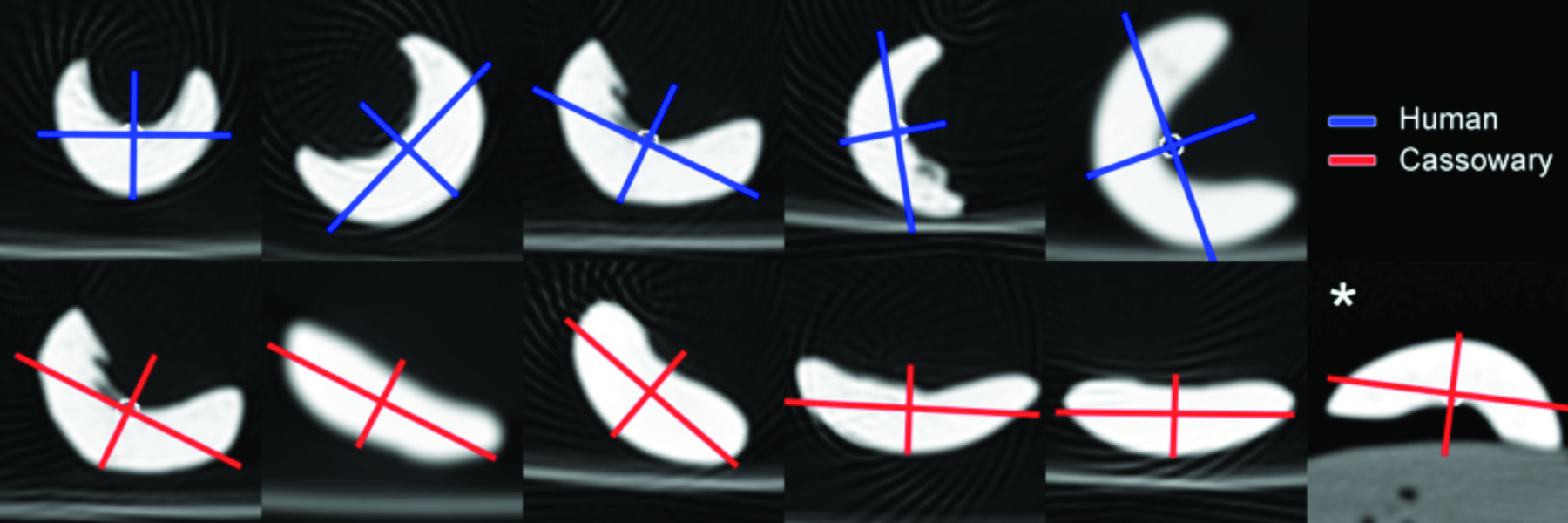

Supplement: Figure S2 [file rsos172067supp2.tif]

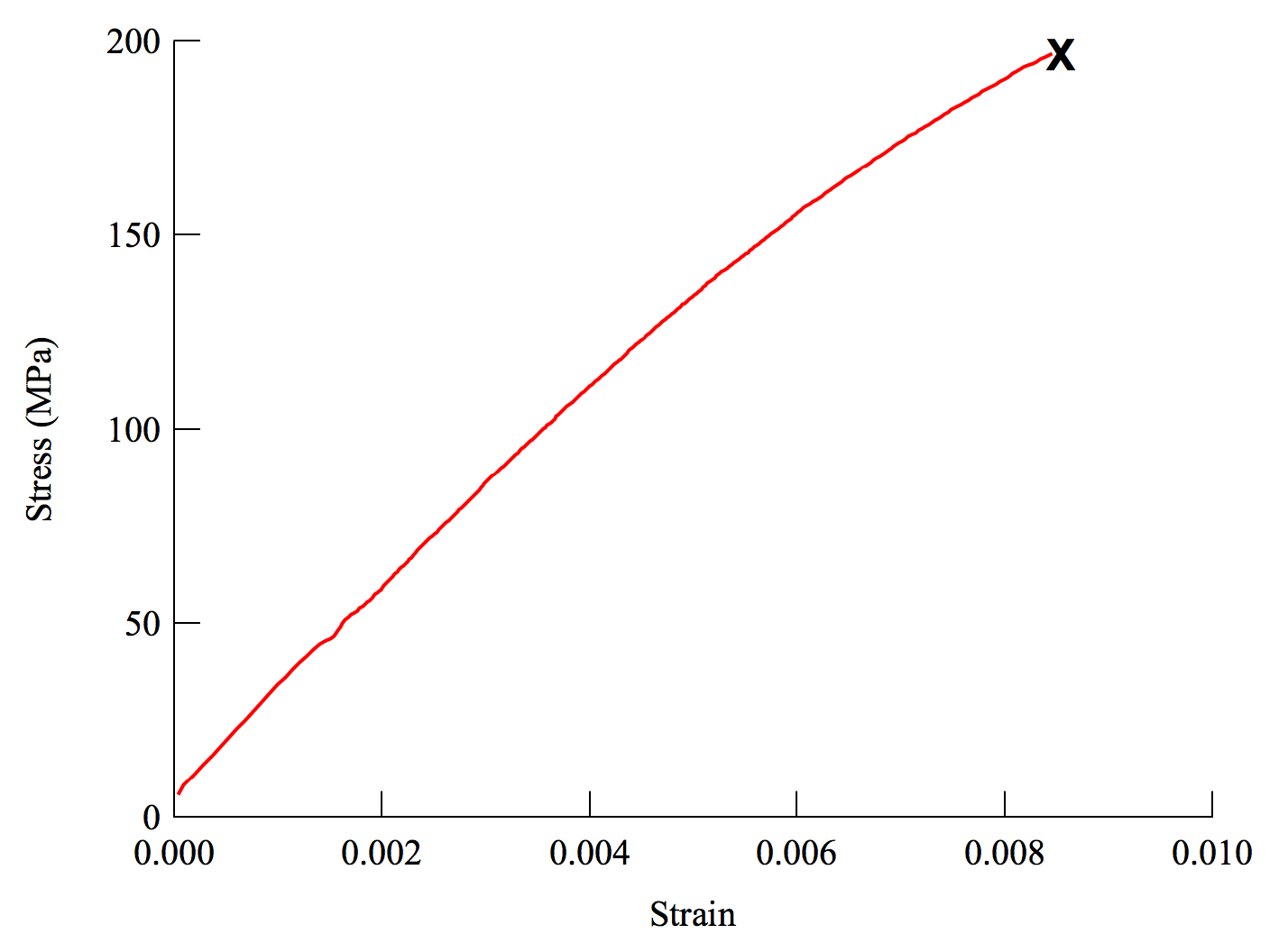

Supplement: Figure S4 [file rsos172067supp4.tif]
